# BLAS3 optimization for the Godson-3B1500

**DOI:** 10.1186/s40064-016-3690-3

**Published:** 2016-11-25

**Authors:** Ming Zhang, Naijie Gu, Kaixin Ren

**Affiliations:** School of Computer Science and Technology, University of Science and Technology of China, 508, Elec-3(Diansan) Building, West Campus of USTC, Huang Shan Road, Hefei, Anhui Province China

**Keywords:** DAE, BLAS, Performance optimization, Godson-3B1500

## Abstract

This paper proposes a performance model for general matrix multiplication (GEMM) on decoupled access/execute (DAE) architecture platforms, in order to guide improvements of the GEMM performance in the Godson-3B1500. This model focuses on the features of access processors (APs) and execute processors (EPs). To reduce the synchronization overhead between APs and EPs, a synchronization module selection mechanism (SMSM) is presented. Furthermore, two optimized algorithms of GEMM for DAE platforms based on the performance model are proposed for ideal performance. In the proposed algorithms, the kernel functions are optimized with single instruction multiple data (SIMD) vector instructions, and the overhead of AP is almost overlapped with EP by taking full advantage of the features of the architecture. Moreover, the synchronization overhead can be reduced according to the SMSM. In the end, the proposed algorithms are tested on the Godson-3B1500. The experimental results demonstrate that the computing performance of dGEMM reaches 91.9% of the theoretical peak performance and that zGEMM can reach 93% of the theoretical peak performance.

## Introduction

Basic linear algebra subprograms (BLAS) (Netlib 2016a) are basic and significant mathematics kernels that provide key functions for high-performance computing (HPC) applications. General matrix multiplication (GEMM), the kernel of level-3 BLAS, is vital for the numerical software Lapack (Netlib 2016b) and performance benchmark Linpack (Netlib 2016c). Especially in Linpack, GEMM accounts for 93% of the entire execution time when it is unoptimized (Zhang et al. 2004). Moreover, GEMM is representative of applications where both computation and memory access are in high demand. Therefore, optimizing the performance of GEMM is significant for guiding improvements in the performance of other applications. Additionally, optimizing computing-intensive applications such as GEMM can simulate potential problems and help to find bugs in newly-developed hardware platforms.

Recently, numerous studies have been conducted to improve the performance of BLAS. Many libraries such as Intel MKL, AMD ACML, ATLAS and GotoBLAS (Goto and Van De Geijn 2008a, b) have been supplied by CPU vendors or HPC researchers. These libraries are aimed at the highest level of performance on various hardware platforms. Additionally, Allen et al. (2009) described auto-tuning and optimized GEMM techniques for GPU. Wang et al. (2013) have presented a template-based optimized framework-AUGEM that can automatically generate fully optimized assembly DLA kernels. The DLA kernels generated by their template-based approach surpass the implementations of MKL and ACML libraries. Moreover, Gu et al. (2008) have conducted much work for BLAS3 optimization on the Godson-2F platform. He et al. (2012) have carried out a study on optimization of BLAS3 on the Godson-3A. Zhang et al. (2012) have released a new library, OpenBLAS, which greatly improves the BLAS3 performance on the Godson-3A.

The optimized algorithms and models described above can efficiently enhance performance and guide users designing optimized frameworks. However, with advancements in the peak computing capability of processors, conventional memory access methods cannot satisfy computational requirements, and traditional optimization methods will be limited. To solve the memory wall, hardware that uses asynchronous memory access technologies has been developed. As a representative, decoupled access/execute architecture (DAE) (Smith 1982, 1984) was proposed by Smith in 1982. Generally, there are several access processors (APs) and execute processors (EPs) on DAE platforms. APs are accountable for memory access, and EPs are responsible for computations. These functional units are independent and can work in parallel. DAE has now become a valued architecture for HPC applications such as BLAS and FFTW due to its superior computing ability and memory access performance.

It is difficult for applications to auto-optimize performance by making full use of EPs and APs, and manual optimizations are needed for the DAE architecture. The Godson-3B series consist of DAE platforms. To improve the performance of applications for the Godson-3B, some studies have been conducted. Zhu (2011) has designed a new algorithm of dGEMM on the Godson-3B, which has been implemented in a simulation platform. Zhao et al. (2013, 2014, 2015) introduced several auto-optimization technologies for BLAS, and the optimizations of dGEMV and dGEMM (ATGEMM) were discussed in detail in the Godson-3B1000. ATGEMM was optimized by using the L2 cache as the intermediate storage space.

However, these studies do not give thorough consideration to the performance impacts of various architectures, including DAE. Therefore, in order to facilitate the optimization of applications in the DAE architecture, a performance model of GEMM for DAE platforms is proposed in this paper. The impacts related to computation and memory access are parameterized in the proposed model, and the time needed by APs and EPs will be evaluated according to the computing account and computing power. The runtime of computing kernels can be preliminarily computed and presented with the features of EPs. Taking into account various factors in the performance model, the overall runtime can be preliminarily computed. The GEMM performance is improved by analyzing the variables that obviously influence the overall runtime in the model. Additionally, several optimized algorithms for ideal performance in the Godson-3B1500 are proposed based on the performance model.

This paper is organized as follows: second section describes the background, including basic GEMM algorithm and the Godson-3B1500. Third section discusses the performance model, followed by the optimization technologies. In fourth section, we detail the proposed algorithms of GEMM. Fifth section presents the correctness of numerical accuracy and the performance improvements. Finally, conclusions are drawn in last section.

## Background

This section describes the background, including the basic GEMM algorithm and the Godson-3B1500. To introduce the Godson-3B1500, we mainly focus on memory access methods and vectorization instructions.

### Basic GEMM algorithm

GEMM is a basic and key algorithm in mathematics. Assuming that the dimensions of the matrix *A*, *B* and *C* are $M\times K$, $K\times N$ and $M\times N$, respectively, GEMM indicates that $C=\alpha C+\beta A\times B$, as shown in (1).

1 $$C_{m,n}=\alpha C_{m,n} + \beta \mathop {\sum }\limits _{k=0}^{K-1}A_{m,k} {\times } B_{k,n}$$

where $A_{m,k}$ denotes the (*m*, *k*) entry of the matrix *A*, $B_{k,n}$ represents the (*k*, *n*) entry of the matrix *B*, and $C_{m,n}$ represents the (*m*, *n*) entry of *C*. For simplification, GEMM mentioned above can be defined as GEMM(*M*, *K*, *N*).

GEMM is called dGEMM when the elements of the matrices are double-precision floating-point numbers. GEMM becomes zGEMM for double-precision floating-point complex numbers. A complex number consists of a real part and an imaginary part. Unlike real numbers, the multiplication of complex numbers consists of four multiplication operations and four addition/subtraction operations. Assuming that complex number *x* is $a+i\times b$ ($i=\sqrt{-1}$), the result is $(ac-bd)+i\times (ad+bc)$ when *x* is multiplied by *c* + *id*. The real and imaginary parts of the complex numbers are stored interleaved in the memory in BLAS.

### Godson-3B1500

**Fig. 1:**
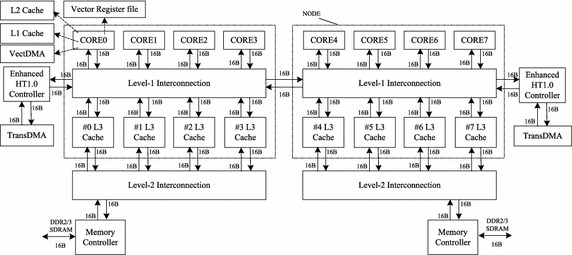
Architecture of the Godson-3B1500

The Godson (Hu et al. 2011) is a family of general-purpose MIPS64 CPU developed at the Institute of Computing Technology, Chinese Academy of Sciences. The Godson-3 Multi-Processor-Chip aims at high-end desktop HPC computers, and the Godson-3B1500 (Hu et al. 2011) is the third generation. As shown in Fig. 1, each CPU consists of two nodes. There are four GS464V cores, one RDMA Matrix Transposition (TransDMA), four 1MB L3 cache, and one DDR3 memory controller in each node. The Godson-3B1500 can issue four instructions in parallel within a single clock cycle, including two floating-point instructions and one memory access instruction.

**Fig. 2:**
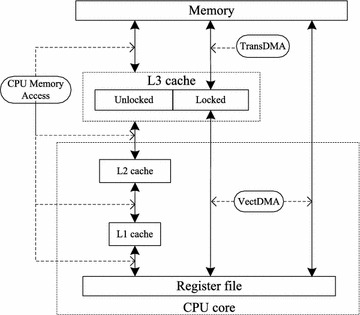
Memory hierarchies and data transfer methods in the Godson-3B1500

As shown in Fig. 2, the memory subsystem of the Godson-3B1500 consists of four storage levels, including the L1 cache, L2 cache, shared L3 cache and memory. Communication between different components in the memory subsystem occurs via the advanced extensible interface (AXI) protocol with cache coherence extension. The L1 and L2 caches of each core are private, while the L3 cache is shared by cores. The caches, which adopt the random replacement strategy, are four-way set-associative, and the size of a cache line is 32B (256-bit) (Gao et al. 2010). Each cache line corresponds to four cache positions, and the four positions are considered as one cache group for simplicity. When the cache missing occurs, the corresponding cache line will be stored into the corresponding cache group. One cache line in the cache group will be chosen by a random algorithm and will be replaced by the new incoming cache line when the cache group is full. Since the random cache replacement strategy does not consider the temporal locality of memory access, the most frequently accessed data may be replaced.

**Fig. 3:**
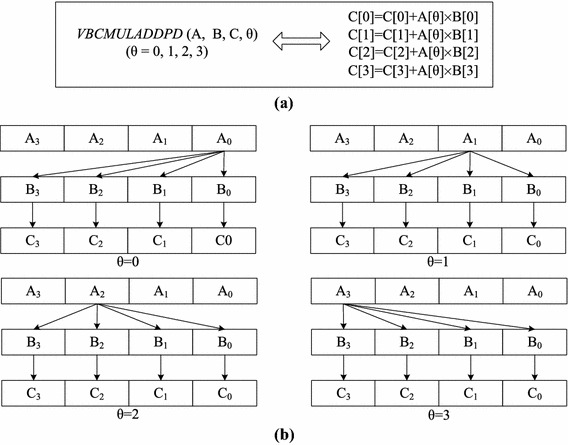
The SIMD vector instruction VBCMULADDPD. **a** Instruction *VBCMULADDPD*. **b** *VBCMULADDPD* operations

As shown in Fig. 2, TransDMA is used to transfer data between the L3 cache and the memory. Meanwhile, VectDMA is used to transfer data between the L3 cache/memory and the vector registers. Each core has one VectDMA that is configured with four channels, *a*, *b*, *c* and *d*. Channels *a*, *b* and *c* transfer data from the L3 cache/memory to vector registers, while channel *d* writes data back to the memory/L3 cache from vector registers. Additionally, each core holds 128-entry vector registers (VectReg) that support single instruction multiple data (SIMD) vector instructions. A vector register can store 256-bit data, such as four double-precision floating-point numbers. The instruction VBCMULADDPD is representative of the SIMD vector instructions in the Godson-3B1500, and it can launch four multiply-add operations of double-precision floating-point numbers in one cycle. As shown in Fig. 3, VBCMULADDPD operates the vector registers *A*, *B*, and *C*, and the results are stored in *C*. The variable $\theta$ represents which number will be operated in vector register *A*.

The Godson-3B1500 can use the mechanism of a cache lock. When the lock window is configured, the cache blocks that are located in the locked L3 cache space cannot be replaced until they are updated manually. For computation-intensive applications, many data need to be accessed and computed multiple times. Cache missing brings numerous extra overheads, which notably influence the performance. It can be even more fatal, especially for platforms with a random cache replacement policy. The lock mechanism can keep frequently required data stored in the locked cache, which can greatly reduce the influence of cache missing for computation-intensive applications and enhance the application performance.

The Godson-3B1500 is a DAE platform. VectDMA and TransDMA can work as APs, and the vector function units work as EPs. The GS464V core can issue two floating-point vector instructions, and each instruction can launch four multiply-add operations in one cycle. There are two floating-point operations in the multiply-add operation. Moreover, there are 8 cores in the EP. When the CPU frequency is 1.5 GHz, the theoretical computing peak capacity ($Perf_{peak}$) can reach $2\times 4\times 2\times 8\times 1.5$ (192.0) GFlops. Generally, the frequency is configured at 800 MHz, and the peak performance is 102.4 GFlops.

## Performance optimizations

These performance optimizations are mainly issued in the MIPS architecture. MIPS is a streamlined and highly scalable reduced instruction set computer (RISC) architecture. It can support SIMD vector instructions and visible pipeline delay slots. There are large number of registers, the number and the character of the instructions in the MIPS. Sometimes it can support different memory access methods such as normal CPU access and DMA access. Generally, there are multilevel caches in the MIPS architectures.

This section presents the performance model of GEMM in DAE architecture. The model is developed according to the features of the DAE architecture and briefly introduces the relationship between overall performance and times of EPs and APs for all DAE architecture. Relationship between performance and architecture, the most important part of the performance model, in which the number of functional units, computational ability of functional units, instruction pipeline structure and capacity of the APs are included, focuses on the Godson-3B1500. Moreover, taking into account the performance model, some optimization technologies are discussed for performance improvements of the GEMM on the Godson-3B1500.

### Performance model

There are two advantages for proposing the performance model for GEMM. The first advantage is to optimize the BLAS3 and guide the designing of algorithms. The second advantage is to offer a modeling method for other applications in the DAE architecture, including Godson-3B1500. The users of Godson-3B1500 can learn how to design an effective algorithm with the help of the GEMM performance model in our manuscript. For a detailed analysis of factors, several variables that represent architecture parameters are defined. These variables are employed into character $T_N$, which is the lower bound for the overall runtime of GEMM(N, N, N). Generally, factors for GEMM performance in DAE architecture include the following parameters.

- $T_{mem}$, which presents the time for data transfer between different storage hierarchies (e.g., memory, caches and register files) by using normal CPU memory access instructions, can be defined as in (2).
  2 $$T_{mem}=\mathop {\sum }\limits _{i=1}^{p}{\mathop {\sum }\limits _{k=1}^{h}{\frac{l(i,k)}{\omega (k)}}} =\mathop {\sum }\limits _{k=1}^h{\frac{L(k)}{\omega (k)}}$$
  where *l*(*j*, *k*) denotes the amount of data that are accessed from the *k*-th layer of memory in the *j*-th computing stage, and *L*(*k*) denotes the total amount of data that are accessed from the *k*-th layer in all computing stages. $\omega (k)$ defines the memory bandwidth of access the *k*-th layer of memory.
- $T_{shuffle}$ It denotes the time for reorganizing the data including changing the position of the data and obtaining the negative of a number in the vector registers. Sometimes, $T_{shuffle}$ can be partially minimized or avoided by optimizing the multiplication of complex numbers and integrating the shuffle function into SIMD vector instructions in the Godson-3B1500.
- $T_{EP}$ It presents the required time for kernel computation of GEMM. The required time mainly depends on the size of GEMM, and the computing capacity of the EPs. For GEMM(*N*, *N*, *N*), $T_{EP}$ can be defined as in (3).
  3 $$T_{EP}=\frac{N^3\times size_{op}}{s\times v \times g \times f} + T_{shuffle} +\lambda _{1}T_{mem}$$
  In (3), $size_{op}$ defines the amount of operations for each operation. *s* defines the number of function units. *v* defines the average degree of parallelism for each instruction. *g* defines the number of operations for each function unit in one cycle. *f* defines the frequency of the CPU. $\lambda _{1}$ denotes the overlapping factor of time for memory access by EPs. The parameters *g*, *s* and *f* are determined by the hardware and they are fixed for the platform.
- ${T_{APi}}$, which presents the time of data transfer for the *i*-th AP, can be defined as in (4).
  4 $$T_{APi}=\mathop {\sum }\limits _{j=1}^p{\frac{Count_{i,j}}{Speed_{AP_{i,j}}}}.$$
  where $Count_{i,j}$ defines the size of data in the *j*-th stage for *i*-th AP. $Speed_{AP_{i,j}}$ denotes the memory access speed of $AP_i$ for the *j*-stage. *p* denotes the amount of stages. There are two stages for the process of GEMM. The first stage is the transfer of data from the memory to the locked L3 cache, and the second stage is the transfer of data from the L3 cache to the vector registers.
- $T_{sync}$ It defines the overhead of the synchronization between APs and EPs, such as the time between computation and DMA in some architectures.
- $T_{extra}$ It denotes the extra overhead of other processes, such as the computation of positions for data prefetching and data storing.

The above-mentioned parameters can be divided into two groups. One group is the time for computation, $T_c$. The other one is the time for memory access, $T_{m}$. $T_c$ can be defined as in (5), and $T_m$ can be defined as in (6).

5 $$T_c= T_{EP} + T_{sync} +T_{extra}$$

6 $$T_m= \max {\{T_{APi}\}} =\max {\left\{ \mathop {\sum }\limits _{j=1}^p{\frac{Count_{i,j}}{Speed_{AP_{i,j}}}}\right\} }$$

The computation and memory access can be processed in parallel in the DAE architecture. The ratio of overlapped memory access time can be defined as $\varrho$. $T_N$ can be derived as in (7). When the time of APs is overlapped with the computational overhead, the performance is mainly determined by the execution overhead. For a large *N*, the extra cost of other processes can be ignored compared with the time of execution, and $T_N$ can be further simplified.

7 $$\begin{aligned} T_N&= \max {\{T_{EP}+T_{sync}+T_{extra},T_m\}} \\&= {T_{EP}+T_{sync}+T_{extra}+(1-\varrho )T_m} \\&= \frac{N^3\times size_{op}}{s\times v \times g \times f} + T_{shuffle} +\lambda _{1}T_{mem} \\&\quad +T_{sync}+(1-\varrho )T_m+T_{extra} \end{aligned}$$

The number of floating-point operations, $N_{cal}$, for GEMM(*M*, *K*, *N*) is fixed. For GEMM(*M*, *K*, *N*), *MKN* operations between matrix elements are needed. There are four multiplications and four additions/subtractions for each operation between matrix elements in zGEMM, and there are one multiplication and one addition in dGEMM. Therefore, $N_{cal}$ is 8*MKN* for zGEMM(*M*, *K*, *N*) and 2*MKN* for dGEMM(*M*, *K*, *N*). The practical performance of GEMM, *P*, can be calculated as $P={N_{cal}}/{T_N}$. After substituting $T_N$ into the formula $P={N_{cal}}/{T_N}$, *P* can be expressed as in (8).

8 $$P=\frac{N_{cal}}{\frac{N^3\times size_{op}}{s\times v \times g \times f} + T_{shuffle} +\lambda _{1}T_{mem} +T_{sync}+(1-\varrho )T_m+T_{extra}}$$

As shown in (8), in order to enhance the performance *P*, the variables $T_{shuffle}$, $T_{sync}$, $T_{extra}$ and $\lambda _{1}$ should be reduced, while $\varrho$ and *v* should be increased. In the DAE architecture, APs and EPs can work in parallel. To reduce the memory access overhead, APs accomplish most missions of memory access, and the normal memory access unit is responsible for the remaining missions of memory access. Most GEMM tasks are computations, and extra overhead makes little difference to the overall runtime. $\varrho$ is influenced by the computation to memory access overhead ratio, and it is mainly determined by the features of the algorithm and hardware. Variables $\varrho$ and $T_{extra}$ will not be discussed in this paper. In the following subsections, the optimizations of *v*, $T_{shuffle}$, $\lambda _{1}$, and $T_{sync}$ are mainly discussed.

### Vectorization

**Fig. 4:**
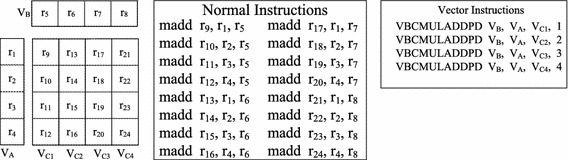
Vectorization for dGEMM computing kernel

**Fig. 5:**
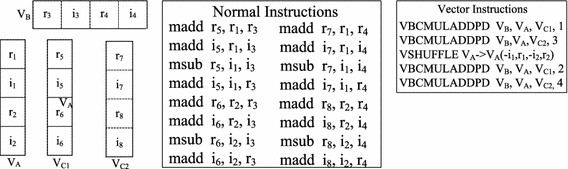
Vectorization for zGEMM computing kernel

The main computational overhead is kernel computation, in which *v* is a decisive factor. *v* presents the average degree of parallelism for each instruction, and vectorization can exponentially increase its value. To improve the overall performance *P*, vectorization is the most important optimization technology to increase *v* theoretically. In GEMM, most computations are multiply-add and multiply-subtract operations. In the Godson-3B1500, the vector instruction VBCMULADDPD can operate the vector registers and launch four multiply-add operations in one cycle. The kernels of GEMM can be vectorized by using vector instructions. Figures 4 and 5 show the methods to optimize GEMM kernels with vector instructions.

Computations of dGEMM are operations between real numbers. All computations are multiply-add operations. Figure 4 shows the operations between matrices *A* and *B*. The sizes of matrices *A* and *B* are $4\times 1$ and $1\times 4$, respectively, for which there exist 16 multiply-add operations. Normal instructions operate the normal registers and can launch 1 operation in one cycle. As shown in Fig. 4, when the normal multiply-add instruction madd is used, there are 16 madd instructions. The original value of *v* equals 1. In the BLAS library, the data in the matrix are arranged in column-major order. When block*B* is preloaded to the L3 cache, matrix transportation is needed to match VectDMA. Compared to the column-major order of the original matrix *B*, the data of block*B* in the L3 cache can be seen in row-major order. Every four neighboring numbers in matrices *A* and *B* can be accessed by the same vector registers. Then, the instruction VBCMULADDPD will be called. At the end of computations, the results are stored into four corresponding vector registers. In total, four vector instructions are needed for kernel computing. After vectorization, the number of kernel instructions will decrease from 16 to 4, and *v* changes from 1 to 4. There are no shuffle operations in dGEMM, and the value of $T_{shuffle}$ is 0.

Computations of zGEMM are operations between complex numbers. Unlike dGEMM, the operations of zGEMM consist of multiply-add and multiply-subtract operations. Figure 5 shows the kernel multiplication of zGEMM(2, 1, 2), and the result is a 2-by-2 matrix. When the kernel is realized with normal instructions, such as the multiply-add instruction madd and multiply-subtract instruction msub, the instructions operate the normal floating-point registers. Normal registers $r_{x}$ and $i_{x}$ are used to store the real and imaginary parts of complex numbers, respectively. As shown in the middle subfigure of Fig. 5, 16 normal instructions are needed for zGEMM(2, 1, 2), and the original value of *v* equals 1.

As shown in the right subfigure of Fig. 5, there are 5 vector instructions to vectorize zGEMM(2, 1, 2). First, the data of matrices *A* and *B* are loaded into vector registers $V_A$ and $V_B$, respectively, by using VectDMA. Then, the results are updated with $V_A$ and the real parts of $V_B$ by calling VBCMULADDPD. Next, the data in the vector register $V_A$ are reorganized and shuffled. The results are updated with $V_A$ and the imaginary parts of $V_B$ by calling VBCMULADDPD in the end. After vectorization, the value of *v* rises from 1 to 4. When the computing kernel is zGEMM(*m*, *k*, *n*), there are $k\times n/2$ shuffle instructions. The total number of vector-computing instructions is $m\times k\times n$. The ratio of the number of shuffle instructions to the number of overall instructions is 1/(2*m* + 1). In other words, shuffle operation takes up approximately 1/(2*m* + 1) of the overall processing time.

### Mechanism for issuing multiple instructions

Assuming that the time of memory access, $T_{mem}$, is fixed, the $\lambda _{1}$ should be reduced to enhance overall performance. The Godson-3B1500 supports the mechanism for issuing multiple instructions, and this mechanism can decrease the $\lambda _{1}$. There are two vector floating-point operation units, two fixed-point operation units, and one memory access unit in each core. Four instructions can be issued simultaneously in one cycle, including two floating-point instructions, one memory access instruction and one fixed-point instruction. In the GEMM kernel, most instructions are computing instructions. To improve the performance, floating-point operation units should be kept working. Non-blocking cache access instructions can be used for data preloading without influencing the efficiency of the computing instruction sequence.

**Fig. 6:**
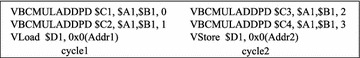
Mechanism for issuing multiple instructions

As shown in Fig. 6, two computation instructions are issued in each cycle, and memory access instructions can be inserted into the instruction sequence. The overheads of memory access are much lower than those of computation, and most of the time of memory access can be concealed by computation. Using a reasonable arrangement of the instruction sequence, the mechanism for issuing multiple instructions can be used to reduce the value of $\lambda _{1}$.

### Synchronization module selection mechanism (SMSM)

As shown in (8), the synchronization overhead, $T_{sync}$, should be reduced to enhance the overall performance. The synchronization overhead between APs and EPs takes up the highest portion of the $T_{sync}$. In order to reduce the synchronization overhead, $\widetilde{R}$, which defines the EP-to-AP time ratio, is proposed. $\widetilde{R}$ can be calculated as in (9), when synchronizations are not considered. When the time of EPs is greater than that of APs, APs can be concealed by EPs. Otherwise, EPs will keep waiting until the APs are completed.

9 $$\widetilde{R}=\frac{T_{EP}+T_{extra}+T_{shuffle}}{\max {\{T_{APi}\}}}$$

It naturally follows that synchronization is needed when EPs have to wait for APs. In the Godson-3B1500, the synchronization module consists of several lines of assembly languages. This module polls the state register for APs circularly, till the value of the register changes to the expected value. In order to reduce the time of synchronization and enhance the performance of GEMM, a SMSM mechanism is proposed. This mechanism deploys the APs, EPs and synchronization module efficiently. The synchronization module is inserted into the computing instruction sequences and takes at least 5 cycles. When EPs have to wait, the overhead of the synchronization module will be larger. If the synchronization module can be discarded, the time of synchronization will be saved, and the performance will be enhanced.

When the time of EPs is less than that of APs, the synchronization module is needed. However, the EP and AP times are not fixed and change slightly with the change of the CPU execution state. If $\widetilde{R}$ is casually calculated and determined, unfavorable scheduling may lead to wrong computing results when the synchronization module is not deployed. To solve this potential problem, $\widetilde{R}$ should be determined cautiously. To ensure correct results, the EP and AP times for kernel computing are tested repeatedly, and the time results are recorded. $\widetilde{R}$ is calculated with the minimal EP time and maximal AP time of the time results. When $\widetilde{R}$ is larger than 1, there is no need for EPs to wait for APs.

The method for calculating the $\widetilde{R}$ is shown below.

- Run the computation kernel for *n* times and test the time.
- Use (9) to calculate the $\widetilde{R}_i$ for the *i*-th test and record the result as $x_i$.
- Calculate the mathematical expectation $\bar{X}$ with $\bar{X}=\frac{1}{n}\mathop {\sum }\nolimits _{i=1}^{n}{x_i}$ and standard deviation $\sigma$ with $\sigma =\sqrt{\mathop {\sum }\nolimits _{i=1}^{n}{(x_i-\bar{X})}^2/n}$.
- Use the one-tailed tests to test whether $\widetilde{R}>1$ (or $\widetilde{R}\le 1$) can be established in 95% confidence level.

According to the definition of $\widetilde{R}$, the SMSM is described as follows. If $\widetilde{R}\le 1$, it is uncertain whether EPs need to wait for APs, or the synchronization module needs to be deployed. Otherwise, the synchronization module can be discarded. In our experiments, the $\widetilde{R}$ can be determined in 95% confidence level when *n* is set to 200.

## Optimized algorithm based on DMA

Classic block matrix multiplications form the essential basis of our algorithms. GEMM consists of multi-level matrix partitions, and every level follows the rules for block matrix multiplication, which are discussed in Goto and Van De Geijn (2008b). When the matrix is divided, there are many small block matrix multiplications. For minor matrix multiplications, the matrix can be divided iteratively. If every matrix partition is correct, the algorithm of GEMM can be proved to be correct.

For *GEMM*(*M*, *K*, *N*), there are three main types of classification:

1. When the matrix A is broken into sub-matrix blocks of dimension *M*-by-$k_0$ and B is divided into sub-matrix blocks of dimension $k_0$-by-*N*, it can be described as $\sum\nolimits _{i=1}^{K/k_0}{GEMM(M,k_0,N)}$.
2. When the matrix B is divided into sub-matrix blocks of dimension *K*-by-$n_0$ and A is not divided, the *GEMM*(*M*, *K*, *N*) can be described as $(GEMM_1(M,K,n_0),\ldots ,GEMM_{N/n_0}(M,K,n_0))$.
3. When the matrix A is divided into sub-matrix blocks of dimension $m_0$-by-*K* and B is not divided, the *GEMM*(*M*, *K*, *N*) can be described as $(GEMM_1(m_0,K,n_0),\ldots ,GEMM_{M/m_0}(m_0,K,N))^T$.

**Fig. 7:**
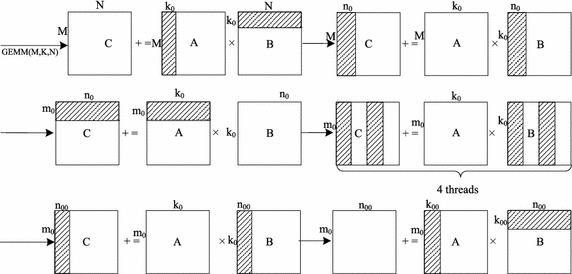
Matrix decomposing methods in the GEMM

As shown in Fig. 7, there are six levels of block matrix multiplications that can be summarized into the above-mentioned types. The first and sixth levels of block matrix multiplication belong to type (1). The second, fourth, and fifth levels of block matrix multiplication belong to type (2). The third one belongs to type (3). TransDMA, VectDMA and non-blocking cache access instructions are responsible for data transfer, and they will not affect the correctness of the algorithms when the correctness of the data transfer is ensured. In total, correct partition procedures lead to correct algorithms.

The ratio of computation to memory access is *N*:4 for basic dGEMM(*N*, *N*, *N*) and *N*:2 for zGEMM(*N*, *N*, *N*). Compared with the computational amount, the amount of memory access is very small. Because of the rapid computational power and slow memory access performance, the memory wall is still the bottleneck of GEMM performance. Many attempts have been made to optimize the BLAS3 with the normal optimization technologies such as loop unrolling, software pipelining or data prefetching of processor. Loop unrolling is used to enhance the re-use of the data in caches to reduce the accounts of memory access. Software pipelining is used to eliminate the correlation between the execution and memory access, and the execution and memory access units can progress in parallel. However, the theoretical peak performance is too high, and the time of memory access of processors cannot be concealed by the execution. Only approximately 35% of the theoretical peak performance can be obtained. Moreover, the parameters of loop unrolling have been adjusted, and the performance is still very low. Therefore, the bottleneck cannot be solved by using normal optimization technologies.

In order to solve the memory wall and guarantee data supply, two novel algorithms based on DAE architecture are proposed, as shown in Algorithms 1 and 2. The computing kernels of these two algorithms utilize optimization technologies such as the vectorization and mechanism for issuing multiple instructions. SMSM is used to reduce the synchronization overhead for these two algorithms. The proposed performance model guides the overall design of algorithms.

### Algorithm 1

As shown in Algorithm 1, there are six loops in GEMM. These loops can be divided into three types, including the outer-loop, middle-loop and kernel-loop. The three loops outside belong to the outer-loop. The fourth loop is in charge of task distribution for multi-threads in the node, and it forms the middle-loop. The remaining loops belong to the kernel-loop. In the first loop, matrices *A* and *B* are divided from the *k*-direction. *A* is broken into sub-matrix blocks of dimension *M*-by-$k_0$, and *B* is divided into sub-matrix blocks of dimension $k_0$-by-*N*.
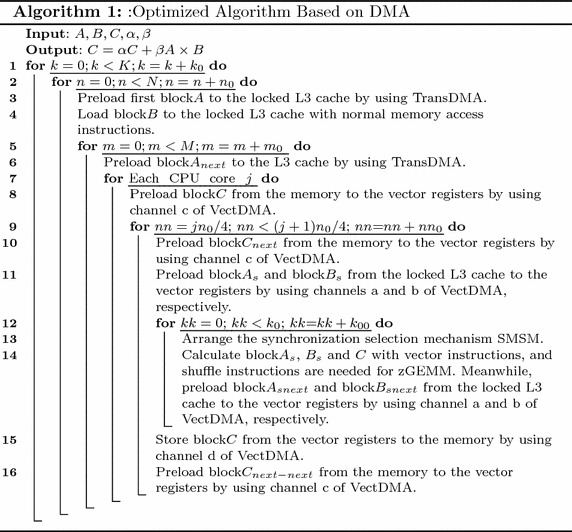


The algorithm mainly discusses the multiplications of *M*-by-$k_0$ block*A* and $k_0$-by-*N* block*B*. First, block*B* is divided with dimensions of $k_0\times n_0$. The processor gives the block*B* access to the L3 cache with normal memory access instructions. At the same time, TransDMA is configured to preload the first $m_0$-by-$k_0$ block*A* to the locked L3 cache. Then, block*B*, which is stored at the locked L3 cache, is successively multiplied by many $m_0$-by-$k_0$ block*A*s in the locked L3 cache. The outer-loop is responsible for data transfer of block*A* and block*B* from the memory to the locked L3 cache. The middle-loop performs an average distribution of block*B* in the locked L3 cache to four threads in the node. Each thread calculates the corresponding $k_{0}$-by-$(n_0/4)$ block*B*.

VectDMA is responsible for data transfer between vector registers and memory/L3 cache. Channels a and b preload block$A_s$ and block$B_s$, respectively. Channel c is responsible for preloading block*C* from the memory to the vector registers, while channel d is in charge of writing block*C* back to the memory. When $m_0$-by-$k_0$ block*A* and $k_0$-by-$n_0$ block*B* are preloaded to the locked L3 cache, the kernel-loop starts to execute. Channel c preloads the $m_0$-by-$n_{00}$ block*C* from the memory to the vector registers. Channel a preloads the $m_0$-by-$k_{00}$ block$A_s$, and channel b preloads the $k_{00}$-by-$n_{00}$ block$B_s$. At the same time, TransDMA starts to preload the next $m_0$-by-$k_0$ block$A_{next}$ to the locked L3 cache. When the computing kernel begins, channel c will preload the next block$C_{next}$ to the vector registers simultaneously.

When the kernel function of multiplication of block$A_s$ and block$B_s$ is called, channels a and b begin to preload the next block$A_{snext}$ and block$B_{snext}$, respectively. After the kernel ends, the computing kernel of the next block$A_{snext}$ and block$B_{snext}$ is called successively. When multiplication of block*A* and block*B* ends, channels d and c begin to write block*C* back and preload the next block$C_{next}$, respectively. At the same time, the multiplication of the next block$A_{next}$ and block$B_{next}$ begins to execute.

The delay of memory access is very long for the Godson-3B1500, and the cache missing rate greatly influences the performance of GEMM. The Godson-3B1500 uses a random cache replacement strategy, and the cache missing rate is significantly higher than those in other strategies for GEMM. For ideal performance, data that are frequently reused should not be replaced from the cache. A mechanism of locking cache is proposed to keep some data in the cache. Experiments demonstrate that if more than half the cache spaces are locked, the system may be paralyzed due to a system deadlock.

In order to ensure that frequently reused data cannot be replaced from the cache, block*A* and block*B* should be stored in the locked cache. When one block*A* is being computed, TransDMA will begin to transfer the next block$A_{next}$. Variable sizeof(xx) is used to define the size of xx. For example, sizeof(matrix element) is utilized to define the size of the matrix element. Two block spaces in L3 cache are assigned to sub-matrix *A*, and block*A* will occupy $2m_0k_0\times$sizeof (matrix element) L3 cache space. Additionally, only one block is assigned to *B*, and *B* will occupy $k_0n_0\times$sizeof(matrix element) L3 cache space. Since only half of the L3 cache can be locked, parameters $m_0, k_0$ and $n_0$ should meet the condition shown in (10).

10 $$(2m_0k_0+k_0n_0)\hbox {sizeof(matrix element)}\le 0.5\times \hbox {sizeof(L3 cache)}$$

In the kernel-loop, the kernel of computation is composed of vector instructions (e.g, multiply-add and shuffle), and the performance of EPs will be maximized. The computing data are stored in the vector registers. Since the size of the vector registers is limited, tiling parameters should be considered. As shown in Algorithm 1, block$A_s$, block$B_s$, and block$C_s$ use the ping-pong processing strategy, and *A*, *B*, and *C* will be assigned two register blocks. Block$C_s$ will occupy the $2m_0n_0\times$sizeof(matrix element) vector register space. To avoid interruption of the instruction pipelining by the performance jitter of VectDMA, several computing groups are needed for *A* and B. Assuming that the number of kernel computation groups is $\rho$, block$A_s$ will occupy $2m_0k_{00}\rho \times$sizeof(matrix element) vector register space, and block$B_s$ will occupy $2k_{00}n_0\rho \times$sizeof(matrix element) vector register space. Parameters $m_0$, $k_{00}$, and $n_0$ should meet the condition shown in (11).

11 $$2((m_0k_{00}+k_{00}n_0)\rho +m_0n_0)\le \frac{ \hbox {sizeof(vector registers)}}{\hbox {sizeof(matrix element)}}$$

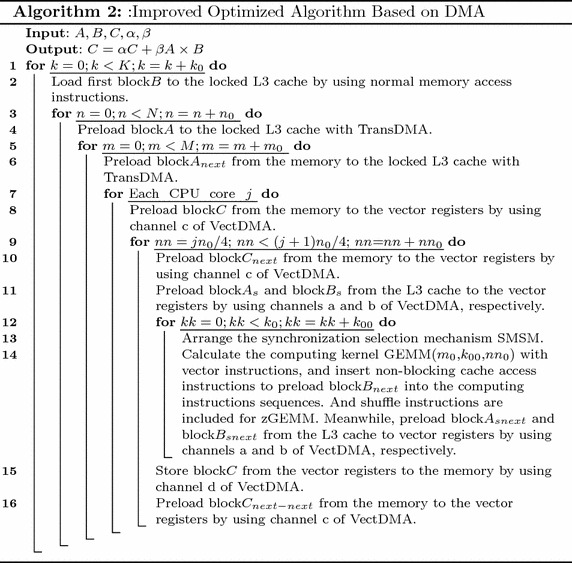


### Algorithm 2

In Algorithm 1, most overheads of memory access are concealed by the computing time. However, the time of loading matrix *B* to the locked L3 cache cannot be concealed. The overhead of loading matrix *B* will influence overall performance. To solve this problem, an optimized algorithm is proposed, in which the time of preloading the matrix *B* can be concealed, as shown in Algorithm 2.

The overhead of data prefetching of block*B* cannot be masked by the EPs in Algorithm 1. It will interrupt the instruction pipelining of the computation kernel to wait for the prefetching of block*B*. As discussed in section “Mechanism for issuing multiple instructions”, the instructions for non-blocking cache access can be inserted into the computational instruction sequence. The locked L3 cache blocks that are assigned to sub-matrix *B* can be divided, on average, into two parts. When one block*A* is being calculated, the next block *A* can preload the data to the other part space. Unlike Algorithm 1, *B* in Algorithm 2 needs two blocks, and *B* will occupy the $2k_0\,\times\,n_0\times$size of (matrix element) L3 cache space. Parameters $m_0$, $k_0$ and $n_0$ should meet the condition shown in (12).

12 $$2 {m_0}\times {k_0} + 2{k_0}\times {n_0} \le \frac{0.5\times \hbox {size\,of\,(L3 cache)}}{\hbox {size\,of\,(matrix element)}}$$

**Fig. 8:**
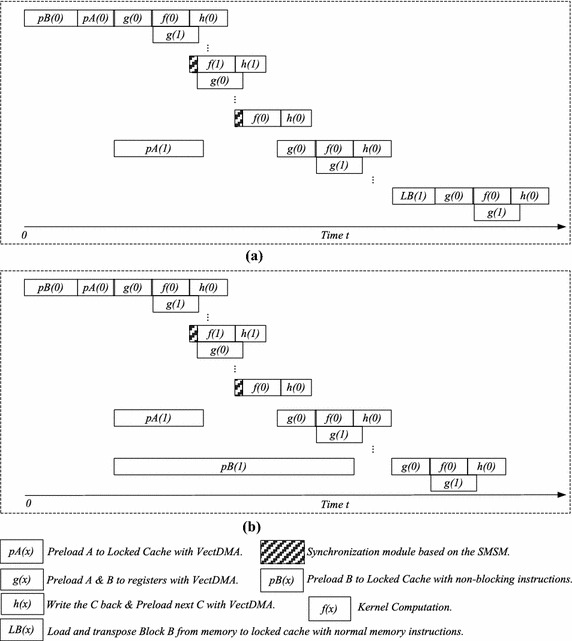
The processes of the proposed algorithms. **a** The process of Algorithm 1, **b** the process of Algorithm 2

The difference between Algorithms 1 and 2 focuses on the method of preloading sub-matrix B. In Algorithm 1, sub-matrix B is loaded using normal memory access instructions, and the computing pipeline will be interrupted to wait for the end of loading B. In Algorithm 2, the non-blocking cache access instructions replace the normal memory access instructions, and the instructions can be inserted into the computing instructions using the mechanism for issuing multiple instructions. Figure 8 shows the process procedures of the algorithms. Most overheads caused by preloading data from the locked L3 cache to the vector registers can be concealed by the computing time in both algorithms. Additionally, the overheads of preloading matrix A to the locked L3 cache can be concealed. Furthermore, as shown in Fig. 8, the overheads of loading sub-matrix *B* in Algorithm 2 can be reduced compared with Algorithm 1.

## Experimental results

To validate the correctness and effectiveness of the proposed algorithms, several experiments were conducted. In this section, we present the experimental testbed and detail the experiments and results.

### Experimental testbed

The kernel functions of the algorithms are mainly implemented in MIPS64 assembly language. The hardware of the testbed is the Godson-3B1500 clocked at 800 MHz. The peak performance of one node is 51.2 GFlops. Experiments were tested on the Loongson-Server Multi-libs system. The software is the GNU Compiler Collection for Godson, and the compile options are “-march=mips64 -mabi=64 -O2”. The compiler supports SIMD vector instructions of the Godson-3B1500. According to (10), (11) and (12), the parameters that produce the best performance are shown in Table 1. VectReg is used to define the number of vector registers used for the algorithms. There are 128-entry 256-bit vector registers. In all, 128 vector registers are used for zGEMM and 120 vector registers are used for dGEMM.

After testing, the ratio of computation to memory access, $\widetilde{R}_{dGEMM}$, equals 0.93 for dGEMM. Compared with dGEMM, zGEMM has a lower memory access ratio ,and its $\widetilde{R}_{zGEMM}$ equals 1.08. According to the SMSM, the synchronization module should be inserted for dGEMM. On the other hand, the synchronization module can be discarded for zGEMM.

**Table 1:** Parameters for GEMM

	*m*_0_	*k*_0_	*n*_0_	*k*_00_	*n*_00_	*ρ*	sizeof(element)	VectReg
*dGEMM* $_{algo1}$	12	512	992	4	12	2	8B	120
*zGEMM* $_{algo1}$	8	512	480	4	8	2	16B	128
*dGEMM* $_{algo2}$	12	512	480	4	12	2	8B	120
*zGEMM* $_{algo2}$	8	512	240	4	8	2	16B	128

### Results analysis

The results are analyzed from two aspects, namely, numerical accuracy and performance. Numerical accuracy analysis is used to verify the correctness and accuracy of the algorithms, while performance analysis is used to calculate the improvements in efficiency of the proposed algorithms.

#### Numerical accuracy

To verify the numerical accuracy, $\delta$ presents the relative error between the correct result and the results of the proposed algorithms. $\delta$ can be computed as in (13).

13 $$\delta = \max \left\{ {\left| \frac{R_{i,j}-R'_{i,j}}{R'_{i,j}}\right| \times 100\%}\right\} , \quad \hbox {i, j} \in [0,N)$$

where $R'_{i,j}$ defines the (*i*, *j*) entry of the correct result and $R_{i,j}$ defines the (*i*, *j*) entry of the results of algorithms to be measured. The precision scope of double-precision floating-point operations is $(-10^{-15},10^{-15})$. $\delta$ should be less than $10^{-15}$ when the precisions of algorithms satisfy the requirements of the original libraries.

**Fig. 9:**
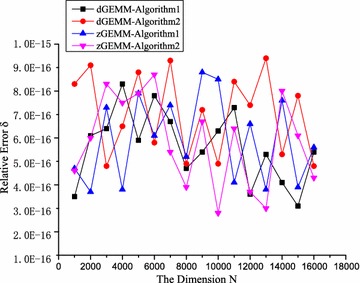
The relative errors $\delta$ for the proposed algorithms

To verify the correctness and numerical accuracy, ATLAS was tested as the reference experiment. The matrix dimension, *N*, ranged from 1000 to 16,000, and experiments were carried out at intervals of 1000. The source data were generated randomly, and the input data of ATLAS and the proposed algorithms were the same. In the zGEMM and dGEMM, the scale of the data of the input matrices makes no difference to the correctness of algorithms, once the results are not out of bounds. The range of the random numbers is set to [−100,000, 100,000], and an uniform distribution is used. After experiments, the relative errors were computed by using formula (13). As shown in Fig. 9, the relative errors of Algorithms 1 and 2 are less than $10^{-15}$. Because ATLAS satisfies the range of errors of double-precision floating-point numbers, the results of ATLAS are set to the standard results. The experiments demonstrate that the results of the proposed algorithms are correct and that the precisions of the proposed algorithms are equivalent to ATLAS.

#### Performance

In the Godson-3B1500, Algorithms 1 and 2 were tested. The algorithm proposed in Zhu (2011) was implemented in a simulation platform (single core) and did not work in the real chips. Therefore, Zhu (2011) was introduced as a representative of studies on the DAE architecture simply and were not tested. For comparison, two standard versions of GEMM, including ATLAS and OpenBLAS, were tested. Moreover, Algorithm 2 without SMSM was tested for zGEMM. These tests were performed on the node (four cores) of Godson-3B1500, where the theoretical computing peak capacity is 51.2 GFlops ($Perf_{peak}$/2).

**Fig. 10:**
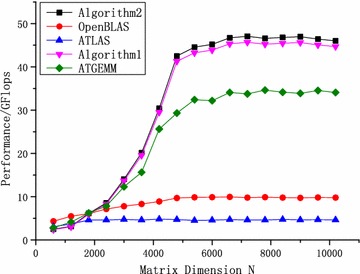
Performance comparison of dGEMM

**Fig. 11:**
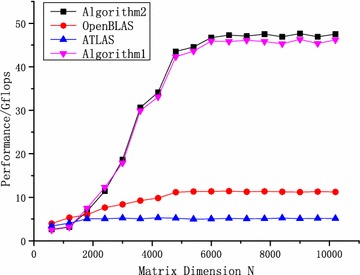
Performance comparison of zGEMM algorithms

One of the most important optimization technologies of ATLAS is data tiling. According to the size of the L1 data cache, the block dimension is $48\times 48$ for dGEMM. The Godson-3B1500 uses the four-way set-associative cache architecture and the random cache replacement strategy. Therefore, there will be many cache conflicts in the matrix blocks when the matrix size is large enough. When a conflict occurs, important data may be replaced out from the L1 cache. Additionally, ATLAS and OpenBLAS cannot fully use the vector instruction set, due to which the computing kernel performs poorly. Only one-fourth of the CPU execution capacity can be used. Moreover, asynchronous data prefetching is not used, and most of the overhead of memory access cannot be concealed. As shown in Figs. 10 and 11, OpenBLAS and ATLAS perform very poorly in the Godson-3B1500.

In Fig. 10, Algorithm 2 performs better than the other algorithms for dGEMM when the size is larger than 2000. When the size is less than 4000, each core has very few tasks, and the power of hardware cannot be exerted. With an increasing size and ratio of computation, optimized algorithms are displaying promising performance. Due to cache missing, there are some oscillations in ATLAS and OpenBLAS with increasing size. However, APs access the data via the locked L3 cache rather than the L2 and L1 caches. The size of GEMM indicates the size of total memory needed in the GEMM. It includes the size of matrices A, B and C. Therefore, the proposed algorithms perform stably, and the performances do not change when the size of dGEMM exceeds the cache size. ATGEMM was optimized by using the L2 cache as the intermediate storage space for Godson-3B. It only optimized the dGEMM for Godson-3B1000, and its optimization methods were not fit for the Godson-3B1500. ATGEMM is an automatic optimized algorithm for dGEMM in the Godson-3B1000 and is optimized by using the L2 cache as the intermediate storage space. Algorithms 1 and 2 are optimized by using the L3 cache as the intermediate storage space. The L3 cache in Godson-3B1500 is twice than the L2 cache in the Godson-3B1000. Moreover, ATGEMM optimized the high levels of the blocking GEMM, and kernel based on the DAE processor was divided into 4 levels. Several levels of the ATGEMM are capable to self-adjust and the parameters are generated by using the automatic optimized algorithm. Manual adjustment to the parameters are needed. The parameters include the main sizes of matrix block for outer loop, the kernel block sizes and other parameters that influence the performance. After the manual adjustments with experience, the performance improves a little. Compared with our approaches, ATGEMM still performs badly. In the Fig. 10, only the performance of original ATGEMM is shown. Moreover, Algorithms 1 and 2 have adjusted the parameters for the Godson-3B1500, and they perform better than ATGEMM in the Godson-3B1500. Compared with Algorithm 1, the time of loading matrix *B* in Algorithm 2 is concealed by using the mechanism for issuing multiple instructions. Algorithm 2 performs approximately 2.5% better when the size is larger than 5200. Since the APs cannot be concealed by the EPs, Algorithm 2 cannot reach the theoretical peak performance. Its best performance is 47.07 GFlops, reaching 91.9% of the theoretical peak.

As shown in Fig. 11, Algorithm 2 performs better than ATLAS and OpenBLAS for zGEMM when the size is larger than 1400. Due to cache missing, there are some oscillations in ATLAS and OpenBLAS with increasing size. However, APs access the data via the locked L3 cache rather than the L2 and L1 caches. Therefore, the proposed algorithms perform stably, and the performances do not change when the size of zGEMM exceeds the cache size. Compared with Algorithm 1, the data preloading of matrix *B* can be concealed by using non-blocking cache access instructions and the mechanism for issuing multiple instructions. Algorithm 2 performs approximately 3.2% better when the size is larger than 6000. In addition, data shuffle is required and cannot be optimized for zGEMM in the Godson-3B1500. The overhead of data shuffle occupies 6% ($1/(2m_0+1)$) of the total runtime. Therefore, Algorithm 2 cannot reach the theoretical peak performance. Its best performance is 47.64 GFlops, reaching 93% of the theoretical computing peak for zGEMM.

**Fig. 12:**
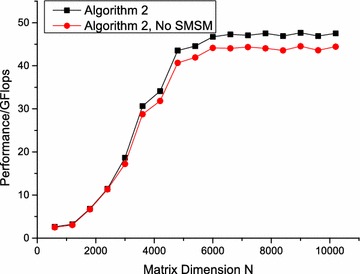
Performance comparison of algorithm 2 for zGEMM

When the overhead of APs can be almost concealed by the EPs, the synchronization module will be redundant for zGEMM. The SMSM can reduce the overhead of the synchronization module and enhance the performance. For zGEMM, the ratio of computation to memory access is large enough, and the synchronization module can be discarded. As shown in Fig. 12, Algorithm 2 without SMSM performs 3% worse than Algorithm 2.

## Conclusion

By virtue of the significance of BLAS, the performance optimization of BLAS has attracted attention from scholars and experts. In this paper, a GEMM performance model for DAE is proposed to analyze the impacts of parameters. Additionally, two optimized algorithms of GEMM are proposed in the Godson-3B1500 based on the performance model. Experiments demonstrate that these two algorithms perform better than other versions of GEMM. The optimized algorithm reaches 93% of the theoretical peak performance for zGEMM and reaches 91.9% of the theoretical peak performance for dGEMM.

However, the performance of GEMM cannot reach the peak performance of the Godson-3B1500. The memory wall is still the bottleneck for HPC applications. It is necessary to investigate how to enhance the performance of memory access in future work. Furthermore, a generic model based on a DAE architecture for BLAS will be designed.
